# Standard anterior temporal lobectomy using a retractorless technique in patients with drug-resistant mesial temporal lobe epilepsy: long-term seizure outcomes and predictors of postoperative seizure freedom

**DOI:** 10.1007/s10143-026-04555-9

**Published:** 2026-11-03

**Authors:** Chonnawee Chaisawasthomrong, Bunpot Sitthinamsuwan, Chottiwat Tansirisithikul, Chumpon Jetjumnong, Sattawut Wongwiangjunt, Vasinee Viarasilpa, Peerada Pongsakornkullachart, Sorawit Viravan, Kanokwan Boonyapisit, Jedsada Thaweepoksomboon, Chanon Ngamsombat, Nasuda Danchaivijitr, Sarun Nunta-aree

**Affiliations:** 1 Faculty of Graduate Studies, Mahidol University, Nakhon Pathom, Thailand; 2 Department of Surgery, Faculty of Medicine Siriraj Hospital, Mahidol University, Bangkok, Thailand; 3 Department of Surgery, Ratchaburi Hospital, Ratchaburi, Thailand; 4 Division of Neurosurgery, Department of Surgery, Faculty of Medicine Siriraj Hospital, Mahidol University, Bangkok, Thailand; 5 Division of Neurosurgery, Department of Surgery, Chiang Mai University, Chiang Mai, Thailand; 6 Division of Neurology, Department of Medicine, Faculty of Medicine Siriraj Hospital, Mahidol University, Bangkok, Thailand; 7 Division of Neurology, Department of Pediatrics, Faculty of Medicine Siriraj Hospital, Mahidol University, Bangkok, Thailand; 8 Department of Internal Medicine, Siriraj Piyamaharajkarun Hospital, Faculty of Medicine Siriraj Hospital, Mahidol University, Bangkok, Thailand; 9 Department of Radiology, Faculty of Medicine Siriraj Hospital, Mahidol University, Bangkok, Thailand

**Keywords:** Drug-resistant epilepsy, Mesial temporal lobe epilepsy, Predictive factor, Retractorless surgery, Seizure-free outcome, Standard anterior temporal lobectomy

## Abstract

Background: Fixed brain retractors risk focal ischemia and cortical injury. Although retractorless dynamic retraction is established in skull base surgery, its role in epilepsy surgery remains poorly characterized. Objective: To evaluate long-term seizure outcomes, safety, and reliability of retractorless standard anterior temporal lobectomy (ATL) in drug-resistant mesial temporal lobe epilepsy (MTLE), and identify predictors of seizure freedom. Methods: We retrospectively analyzed 42 patients who underwent retractorless standard ATL for drug-resistant MTLE. Dynamic retraction used bipolar forceps and pressure-controlled suction. The primary outcome was seizure freedom (Engel Class Ia) at minimum 24-month follow-up; secondary outcomes were complications and resection adequacy on postoperative magnetic resonance imaging (MRI). Predictors were identified using multivariable logistic regression and Cox proportional hazards models. Results: At a mean follow-up of 66.3 months, 69% of patients achieved complete seizure freedom (Engel Class Ia), and 83.3% achieved freedom from disabling seizures (Engel Class I). Postoperative MRI confirmed adequate resection in 95.2% of cases. Complications were uncommon and unrelated to retraction injury. Low preoperative seizure frequency (< 3 seizures/week) was the sole independent predictor of seizure freedom (odds ratio, 24.21; *P* = 0.026), while higher frequency (≥ 3 seizures/week) was associated with increased recurrence risk (hazard ratio, 7.34; *P* = 0.042). Conclusion: Retractorless standard ATL is safe and effective for drug-resistant MTLE, achieving long-term seizure control while minimizing retraction-associated risks. Preoperative seizure burden was the dominant prognostic marker, supporting early surgical intervention.

**Supplementary Information:**

The online version contains supplementary material available at https://doi.org/10.1007/s10143-026-04555-9.

## Introduction

Epilepsy is one of the most common and disabling neurological disorders, affecting patients of all ages worldwide [1–4]. Approximately 20%–30% of patients exhibit pharmacoresistance and fail to achieve seizure control with medication alone [5–9]. Epilepsy surgery is a major treatment strategy used to control intractable seizures, reduce seizure burden, improve quality of life, and address psychosocial sequelae [10–13].

Mesial temporal lobe epilepsy (MTLE) is a spectrum of epilepsy syndromes in which epileptogenic activity arises from the mesial temporal structures, including the hippocampus, amygdala, and parahippocampal gyrus. It is one of the most common surgically remediable epilepsy syndromes worldwide [14]. Standard anterior temporal lobectomy (ATL) remains the most widely performed operation for MTLE. The procedure entails removal of the anterolateral temporal neocortex together with resection of the anterior hippocampus, parahippocampal gyrus, and amygdala [15].

During neurosurgical procedures used to access deep-seated brain lesions, brain retractors are commonly used to improve visualization, stability, and maneuverability and to reduce surgeon fatigue. However, excessive brain retraction may induce cerebral injury, resulting in postoperative neurological deficits [16, 17]. These adverse events are attributed to local ischemia [18–20] or direct mechanical trauma [20, 21]. Novel instruments, technical refinements, and quantitative methods have been developed to mitigate brain retraction injury [22–25]. These advances have facilitated the adoption of retractorless techniques for the management of intracranial lesions [21, 26–30], with reported outcomes superior to those of traditional retraction-based methods [31, 32].

Nevertheless, fixed retractors remain commonly used in epilepsy surgery, including ATL for MTLE, potentially increasing the risk of cortical injury and compromising seizure control. To date, evidence supporting the retractorless approach in the specific context of epilepsy surgery is limited. We therefore conducted this study to evaluate the long-term seizure outcome, safety, and reliability of standard ATL performed with a retractorless technique in patients with drug-resistant MTLE. Predictors of complete postoperative seizure freedom were also investigated.

## Materials and methods

### Patient population

This retrospective cohort study enrolled patients with drug-resistant MTLE who underwent retractorless standard ATL with postoperative follow-up a​t Faculty of Medicine Siriraj Hospital, Mahidol University, between January 2013 and September 2025. Drug-resistant epilepsy was defined as failure of at least 2 antiseizure medications, used at adequate doses and appropriate for the seizure type, to provide effective seizure control.

Eligible patients were preoperatively evaluated by a multidisciplinary team. Presurgical evaluation comprised clinical assessment, seizure semiology, scalp electroencephalography (EEG), high-resolution magnetic resonance imaging (MRI), single-photon emission computed tomography (SPECT), and the Wada test. Retractorless standard ATL was selected by the surgeon’s preference and proficiency. All included patients had complete medical records and a minimum postoperative follow-up of 24 months. Patients were excluded if they had incomplete data, follow-up of less than 24 months, or had undergone procedures involving additional extratemporal resection.

### Ethical approval

This study was approved by the Siriraj Institutional Review Board, Faculty of Medicine Siriraj Hospital, Bangkok, Thailand (Si-920/2025). The study was conducted in full compliance with international guidelines for the protection of human research participants. These guidelines included the Declaration of Helsinki, the Belmont Report, the CIOMS Guidelines, and the International Conference on Harmonization Good Clinical Practice (ICH-GCP).

### Data collection

The data were retrieved from the electronic database as a case record form for the study. They are summarized below:

- **Demographic characteristics**: age, sex, preoperative duration of epilepsy, preoperative diagnosis, location of the lesion on MRI, and risk factors for epilepsy.
- **Presurgical evaluation data**: seizure semiology, interictal and ictal epileptiform discharges on scalp EEG, lesion characteristics on cranial MRI, interictal SPECT, Wada test results, and preoperative antiseizure drugs (ASDs).
- **Intraoperative data**: side of surgery, operative time, and intraoperative blood loss.
- **Postoperative data**: complications, length of hospital stay, postoperative cranial MRI, need for a second operation, postoperative ASD regimen, and duration of postoperative follow-up.
- **Pathological data**: pathological diagnosis and the histopathology of the hippocampus, amygdala, and anterior temporal cortex.

### Outcome assessment for retractorless standard ATL

Postoperative seizure outcome was assessed using the Engel classification. Patients were stratified into 2 groups: a seizure-free group (Engel Class Ia, defined as completely seizure-free) and a non–seizure-free group (Engel Class Ib–IV).

Complications were assessed and diagnosed objectively based on clinical assessments during postoperative admission, follow-up visits, and postoperative investigations. These complications included immediate postoperative convulsions, electrolyte imbalance, postoperative infection, neurological deficits, and radiographic abnormalities associated with the surgical procedure. Evidence of surgery-related brain insult was assessed by early postoperative cranial CT, looking for cerebral edema, cerebral contusion or hematoma, and venous infarction.

**Fig. 1:**
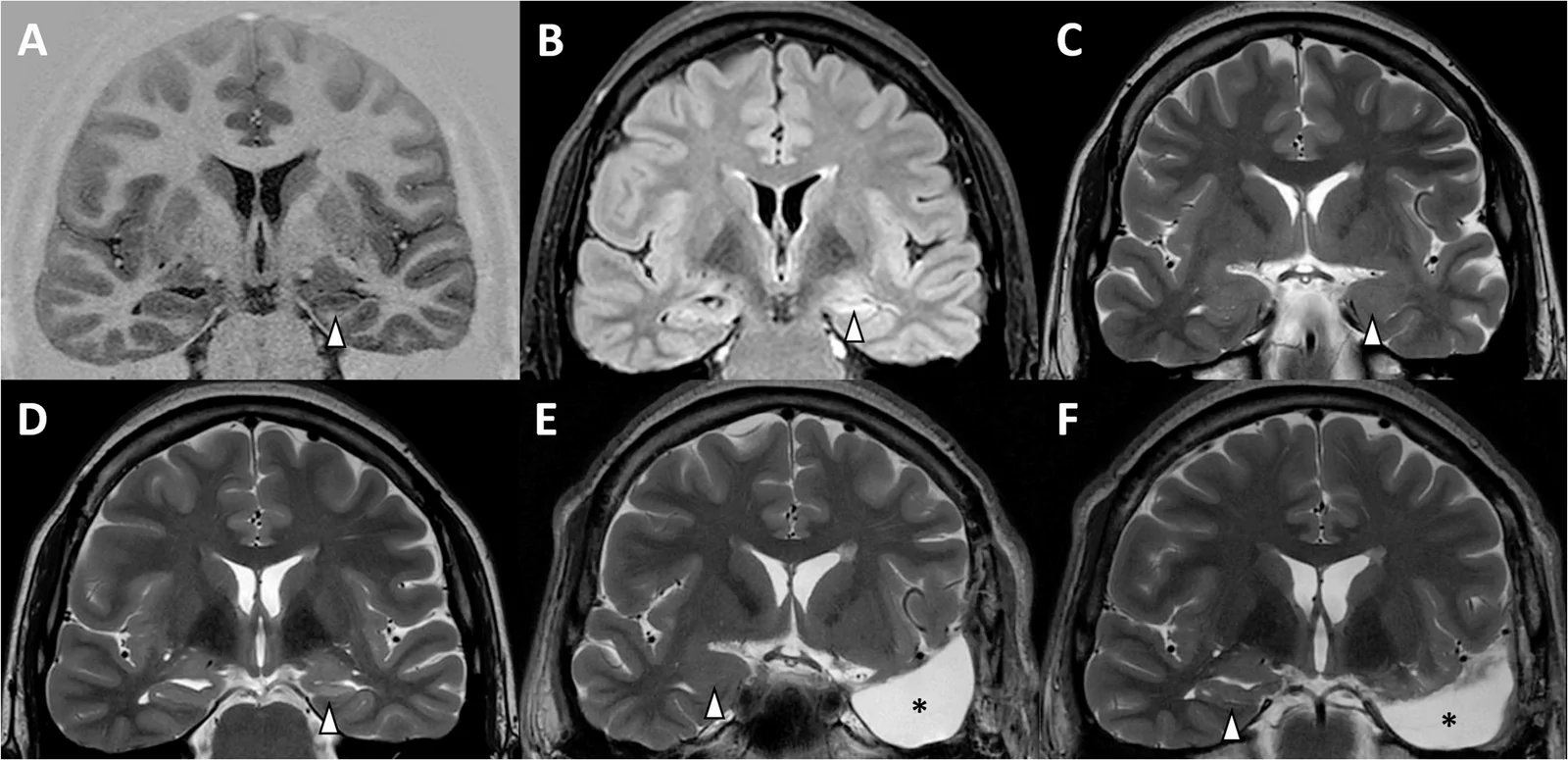
Preoperative and postoperative coronal magnetic resonance imaging (MRI) in a patient with hippocampal sclerosis who underwent retractorless standard anterior temporal lobectomy. (**A**) Preoperative inversion-recovery sequence showing an atrophic left hippocampus (arrowhead). (**B**) Preoperative fluid-attenuated inversion recovery (FLAIR) sequence showing a hyperintense left hippocampus (arrowhead). (**C**) Preoperative T2-weighted MRI showing a coronal section through the left amygdala (arrowhead) before resection. (**D**) Preoperative T2-weighted MRI showing a small left hippocampus (arrowhead). (**E**) Postoperative T2-weighted MRI in the same section as (**C**) showing the right amygdala (arrowhead) and the resection cavity (asterisk) of the left amygdala and temporal cortex. (**F**) Postoperative T2-weighted MRI in the same section as (**D**) showing the right hippocampus (arrowhead) and the resection cavity (asterisk) of the left hippocampus and temporal cortex. Abbreviations: FLAIR, fluid-attenuated inversion recovery; MRI, magnetic resonance imaging

The adequacy of retractorless standard ATL was assessed using postoperative high-resolution cranial MRI (Fig. 1), which was performed in all cases to evaluate the extent of resection and any complications.

Adequate resection of the anterior temporal neocortex was defined as resection extending 4–4.5 cm on the dominant language side or 5–5.5 cm on the non-dominant language side. The resection was measured from the anterior tip of the middle cranial (temporal) fossa to the posterior resection margin of the cortex.

Adequate resection of the mesial temporal structures was defined as resection of the anterior 2.5–3 cm of the hippocampus and adjacent parahippocampal gyrus, together with complete resection of the amygdala.

### Surgical procedures

**Fig. 2:**
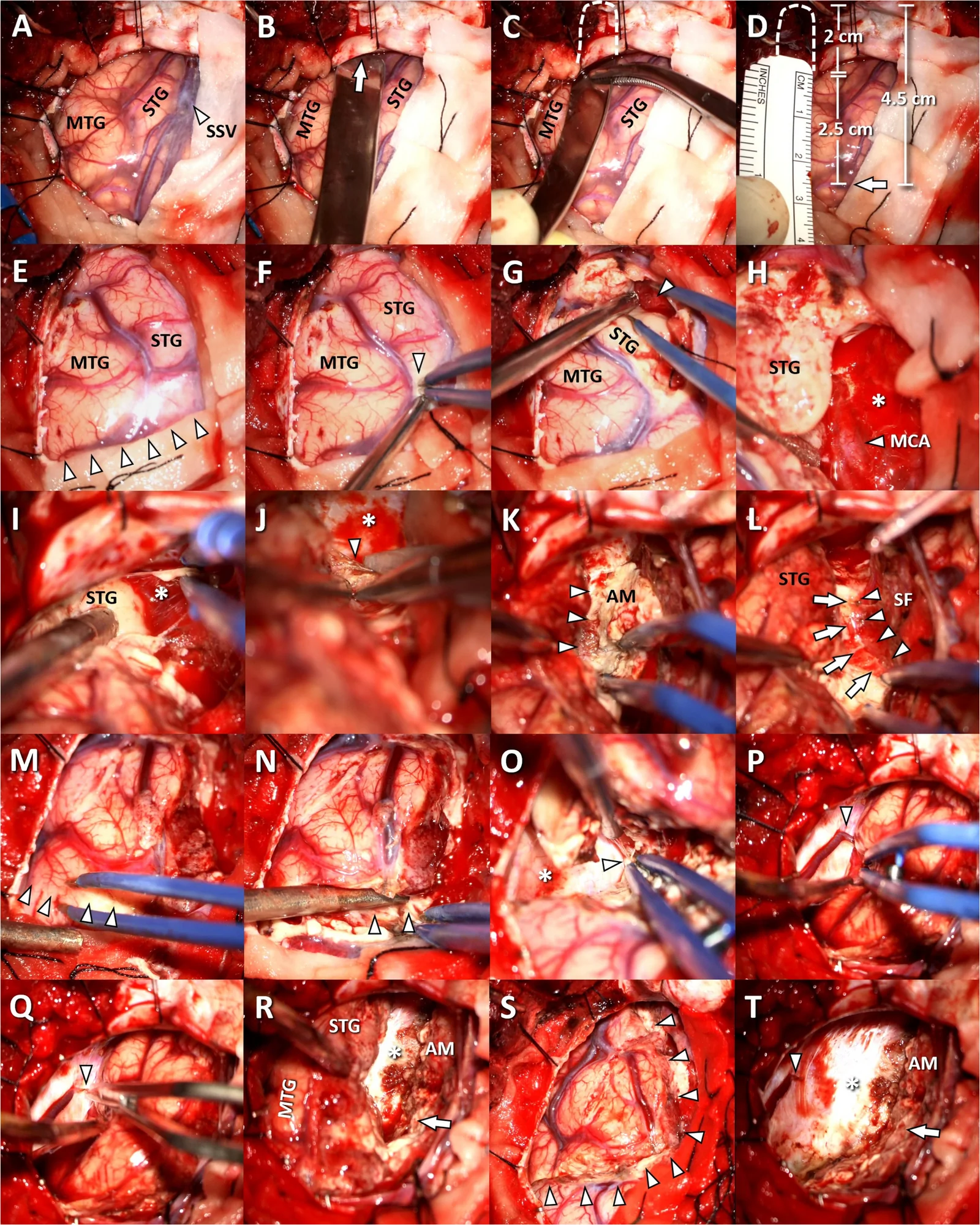
Resection of the anterior temporal cortex, left side. (**A**) Exposure of the superior and middle temporal gyri and superficial Sylvian vein following dural opening. (**B**) A brain spatula is inserted under the dura mater along the middle temporal gyrus toward the anterior temporal tip (arrow), in order to measure the cortex hidden beneath the dura mater. (**C**) Use of an arterial clamp for marking the length of temporal cortex under the dura mater (dotted line). (**D**) Use of a ruler for measuring the length of the exposed temporal cortex, a summation of both lengths of the temporal cortex being the total length of the temporal cortex that will be resected. For example, the length of the temporal cortex under the dura mater (dotted line) is 2 cm, and that of the exposed temporal cortex is 2.5 cm; the summation is 4.5 cm from the anterior temporal tip, which indicates the posterior resection margin (arrow). (**E**) A long cottonoid sheet is placed to mark the posterior boundary of the cortical resection (multiple arrowheads). (**F**) Cortical resection begins with coagulation of the superior temporal gyrus (arrowhead). (**G**) Subpial resection of the superior temporal gyrus revealing the arachnoid mater covering the Sylvian fissure (arrowhead). (**H**) Further subpial removal of the superior temporal gyrus showing the arachnoid mater (asterisk) covering the middle cerebral artery. (**I**) Subpial resection of the superior temporal gyrus from the arachnoid mater (asterisk) at the anterior temporal tip. (**J**) The leptomeninges of the inferior temporal cortex (arrowhead) are coagulated and cut, revealing the dura mater of the middle cranial fossa (asterisk). (**K**) The temporal cortex is disconnected (multiple arrowheads) from the amygdala. The amygdala is kept for the last resection. (**L**) Moving posteriorly, the superior temporal gyrus is dissected away from the Sylvian fissure until the inferior circular sulcus of the insula (multiple arrowheads) and temporal stem (multiple arrows) are observed. (**M**) Starting posterior disconnection by coagulation of the temporal cortex along the line of posterior resection margin (multiple arrowheads). (**N**) Deeper resection in the coronal plane along the posterior resection margin (arrowheads). (**O**) The leptomeninges of the basal temporal cortex are coagulated and cut (arrowhead) along the posterior resection margin showing the dura mater of the middle cranial fossa (asterisk). (**P**) Care should be taken to identify the temporal bridging vein (arrowhead) along all cortical surfaces. (**Q**) The bridging vein connecting between the resected temporal cortex and dura mater is coagulated and cut (arrowhead). (**R**) Medially, the temporal cortex is disconnected from the amygdala and temporal white matter (arrow) covering the hippocampus. The middle cranial fossa (asterisk) is clearly exposed. (**S**) After complete cortical disconnection, the outline of the temporal cortex resection is demonstrated (multiple arrowheads). (**T**) After removal of the anterior temporal cortex, the amygdala, temporal white matter (arrow) covering the hippocampus, disconnected bridging vein (arrowhead), and middle cranial fossa (asterisk) are visualized. Abbreviations: AM, amygdala; MCA, middle cerebral artery; MTG, middle temporal gyrus; SF, Sylvian fissure; SSV, superficial Sylvian vein; STG, superior temporal gyrus

**Fig. 3:**
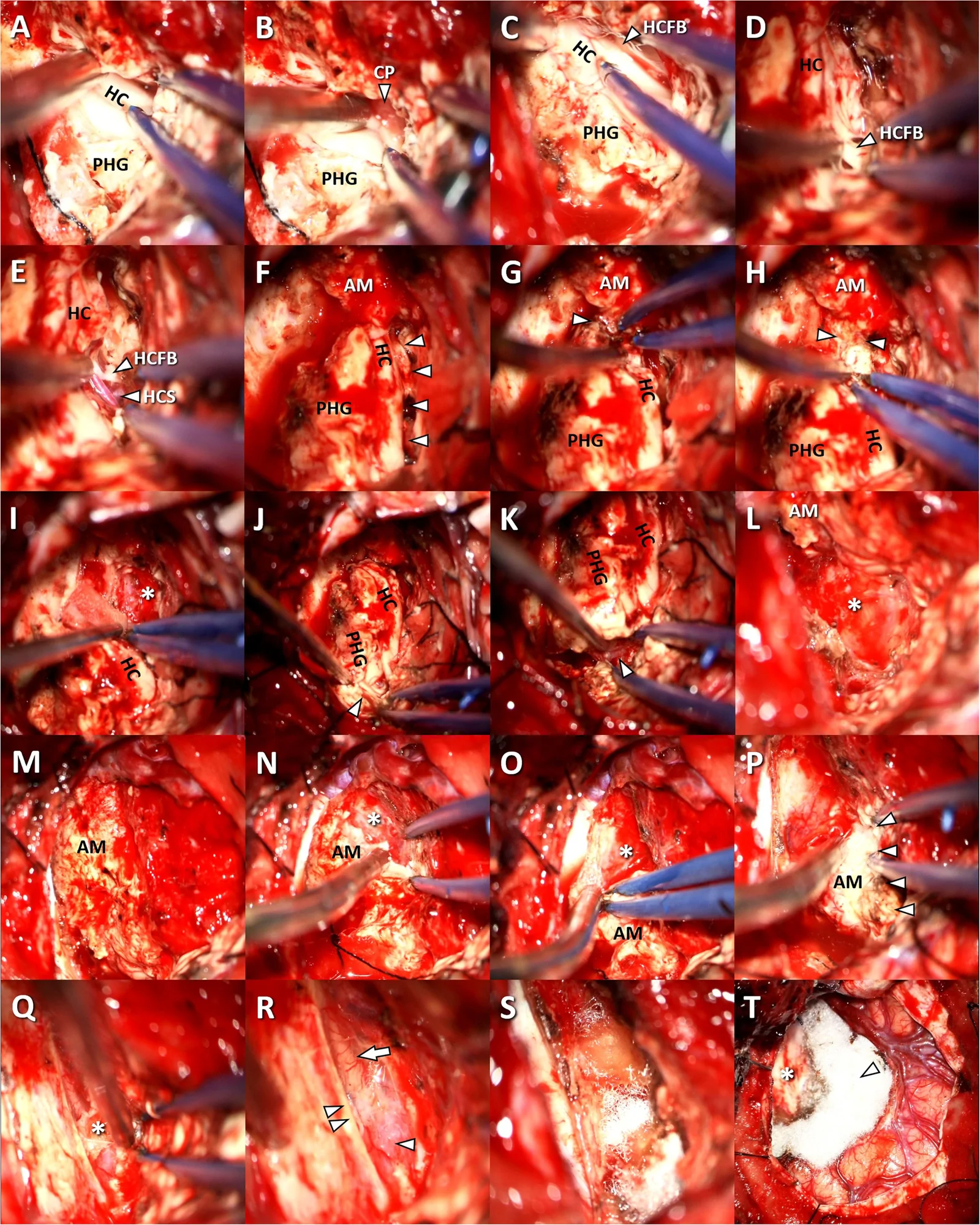
Resection of the hippocampus and parahippocampal gyrus, left side,​ panels (**A**–**L**); amygdalectomy, left side, panels (**M**–**T**). (**A**) After removal of the temporal white matter covering the temporal horn of the lateral ventricle, the hippocampus and parahippocampal gyrus are exposed. (**B**) The choroid plexus is identified in the temporal horn. (**C**) The hippocampal fimbria is found medial to the hippocampus. (**D**) The hippocampal fimbria is meticulously removed. (**E**) After disconnection of the hippocampal fimbria, the hippocampal sulcus and arterial branches supplying the hippocampus are exposed. (**F**) Exposure of the outline of hippocampal fimbria resection (multiple arrowheads) and hippocampus, which anteromedially connects to the amygdala. (**G**) Starting disconnection between the hippocampal head and amygdala by coagulation at their connection (arrowhead). (**H**) Complete disconnection (arrowheads) between the hippocampal head and amygdala. (**I**) The hippocampus is peeled out from the arachnoid mater covering the perimesencephalic cistern (asterisk). (**J**) Posterior disconnection of the hippocampus and parahippocampal gyrus (arrowhead) in an appropriate length from the hippocampal head. (**K**) Deeper disconnection showing the arachnoid mater and adjacent arterial branches to the hippocampus (arrowhead). (**L**) After removal of the hippocampus and parahippocampal gyrus, the arachnoid mater covering the perimesencephalic cistern (asterisk) is observed. The amygdala is preserved for the final step of resection. (**M**) The amygdala is identified anterior to the area of the resected hippocampus. (**N**) The amygdala is subpially dissected away from the adjacent arachnoid mater (asterisk). (**O**) The amygdala is peeled out from the medial arachnoid mater (asterisk). (**P**) There is no identifiable plane between the amygdala and the overlying brain tissue. The superior boundary of amygdalar resection is therefore kept along an imaginary line connecting the inferior choroidal point and the limen insulae; resection must not extend superior to this line (multiple arrowheads). (**Q**) The residual amygdalar tissue is gradually removed using pressure-controlled suction. The medial arachnoid (asterisk) is preserved during resection to avoid injury to the underlying neurovascular structures. (**R**) The authors prefer removing the amygdala in an almond-shaped contour. After a complete amygdalectomy, the medial edge of the tentorium cerebelli (double arrowheads) and the oculomotor nerve (arrow) and posterior cerebral artery (arrowhead) beneath the arachnoid mater can be clearly seen. (**S**) Hemostatic agent is applied on surgical surfaces. (**T**) Exposure of the resection area showing the middle cranial fossa (asterisk) and hemostatic agent (arrowhead) applied on the resection areas. Abbreviations: AM, amygdala; CP, choroid plexus; HC, hippocampus; HCFB, hippocampal fimbria; HCS, hippocampal sulcus; PHG, parahippocampal gyrus

All operations were performed by a single surgeon (B.S.). A retractorless microsurgical technique was used, with an operating microscope and dynamic retraction provided by bipolar forceps and pressure-controlled suction. The procedure is described below (Supplementary Fig. 1) (Figs. 2 and 3).

- **Patient positioning**: After general anesthesia and endotracheal intubation, the patient was placed supine on the operating table. The head was rotated approximately 30° to the contralateral side and fixed in a Mayfield head holder, with the ipsilateral shoulder elevated using a surgical cushion. The head was also extended 10° to improve access to the temporal fossa, the temporal horn of the lateral ventricle, and the hippocampus. This head orientation effectively utilized gravity to allow the temporal lobe to fall naturally away from the floor of the middle cranial fossa.
- **Surgical incision**: A question-mark incision was started within the hairline anterior to the tragus, extending superoposteriorly above the pinna and then curving anteriorly along the superior temporal line. The superficial temporal artery, where it crossed the lower part of the incision, was dissected free and preserved. The temporalis fascia and muscle were incised along the line of the skin incision. The myocutaneous flap was dissected and reflected anteroinferiorly to expose the temporal skull. Inferior dissection of the temporalis muscle had to be sufficient to allow palpation of the zygomatic arch. After complete soft-tissue dissection, the temporal skull was prepared for craniotomy.
- **Craniotomy**: A standard temporal craniotomy was performed for the ATL. The craniotomy size had to be appropriate for the individual patient to expose the superficial Sylvian vein and to facilitate temporal cortex resection. Three burr holes were made with a high-speed drill at the frontal keyhole, the inferior temporal region, and the superior temporal line. The temporal bone was cut between these burr holes to create a free bone flap, which was carefully dissected from the dura mater and removed. Residual temporal bone toward the anterior tip of the temporal lobe and the temporal fossa was removed using an angled bone rongeur. This additional bone removal markedly improved visualization of the temporal cortex beneath the skull. The superficial sphenoid ridge was also removed with a smaller bone rongeur to improve access to the anterior tip of the temporal lobe. After craniotomy, complete hemostasis was achieved before opening the dura mater. The petrous temporal bone air cells were always inspected and, if exposed, were waxed or packed to prevent cerebrospinal fluid leak.
- **Dural incision**: A U-shaped durotomy was routinely used, with the base reflected anteriorly. Bleeding dural vessels were coagulated, and the dural flap was tacked to the temporalis muscle. After the durotomy, the superficial Sylvian vein, the superior and middle temporal gyri, and the cortical vessels should be clearly visualized.
- **Identification of the posterior resection margin**: Before starting resection of the anterior temporal cortex, the key anatomical landmarks were identified, including the superficial Sylvian vein, the Sylvian fissure, and the superior and middle temporal gyri. These structures guided the resection. The posterior margin was always measured, and the operating microscope was introduced at this step. A brain spatula was inserted under the dura mater along the middle temporal gyrus toward the anterior temporal pole. The length of temporal cortex under the dura mater was measured and added to the length of the exposed cortex to determine the posterior resection margin. The length of anterior temporal resection, measured from the temporal pole to the posterior resection margin along the middle temporal gyrus, was 4.5 cm in the dominant language hemisphere or 5.5 cm in the non-dominant language hemisphere. Accurate marking of the resection margin was essential to avoid posterior injury to eloquent cortex (the primary auditory and language receptive areas) and to the temporal optic radiation.
- **Anterior temporal cortex resection**: Before cortical resection, an elongated cottonoid sheet was placed over the superficial Sylvian vein to prevent inadvertent injury. Cortical resection began on the superior temporal gyrus. Bipolar coagulation was performed along the superior temporal gyrus from the posterior resection margin toward the anterior temporal pole. Subpial resection with pressure-controlled suction was used to dissect the superior temporal gyrus from the superficial Sylvian vein and the arachnoid covering the Sylvian fissure. The arachnoid plane over the fissure was preserved in its entirety, allowing the middle cerebral artery and its branches to be visualized and protected beneath the membrane. Bleeding points on the arachnoid were meticulously coagulated, and hemostatic agents, such as absorbable oxidized cellulose, were applied as needed. The anterior temporal cortex was completely dissected from the Sylvian fissure and its contents. The dissection then continued inferiorly to the inferior circular sulcus of the insula, which served as a landmark for temporal stem (white matter) resection.

We then turned to the posterior boundary, repositioning the microscope toward the temporal fossa. The temporal cortex at the posterior resection margin was coagulated in the coronal plane, from the superior temporal gyrus downward to the middle and inferior temporal gyri. Because the inferior temporal and fusiform gyri lay hidden under the dura mater, repositioning the operating view markedly improved their visualization. Subpial removal of brain tissue then proceeded carefully, with attention to bleeding from sulcal vessels, until the fusiform gyrus was posteriorly disconnected. Returning to the temporal stem, the temporal white matter was removed gradually until the temporal horn of the lateral ventricle was reached. The temporal horn was the most important surgical landmark, leading to identification of the hippocampus and amygdala. Cerebrospinal fluid was often encountered as clear fluid escaping from the ventricle, with the hippocampus visible within it. The white matter covering the temporal horn was removed to expose an adequate length of the hippocampus (the head and anterior body).

The anterior temporal neocortex and its basal leptomeninges were then disconnected from the mesial temporal structures in an anterior-to-posterior direction. Particular care was taken during disconnection of the anterior temporal pole to avoid injury to the main insertion of the superficial Sylvian vein and the adjacent sphenoparietal sinus. The uncal tissue, hippocampus, and parahippocampal gyrus were preserved for the subsequent mesial temporal resection. All cortical bridging veins of the anterior temporal cortex were identified and coagulated before removal of the lobe. The anterior temporal cortex was finally removed en bloc, the specimen comprising the superior, middle, and inferior temporal gyri together with the fusiform gyrus. Resection was performed using only a bipolar coagulator and pressure-controlled suction. When required, 2 suction devices were used—1 for suction and the other for suction with counter-traction. No neuronavigation system, electrocorticography, or ultrasonic aspirator was used during the resection.

- **Hippocampal and parahippocampal resection**: En bloc resection of the hippocampal head, the anterior hippocampal body, and the parahippocampal gyrus was planned. Returning to the exposed temporal horn of the lateral ventricle, the choroid plexus and the inferior choroidal point were identified within the ventricle. The inferior choroidal point was a crucial landmark for resection of the mesial temporal structures. It lay at the inferior end of the choroid plexus attachment, just posterior to the hippocampal head. The hippocampal fimbria was then gently incised to expose the hippocampal sulcus. Identification of the hippocampal sulcus was useful for gauging the depth of hippocampal resection. Within the sulcus, arterial feeders from the posterior cerebral artery, such as the hippocampal artery, were carefully coagulated and divided.

Anteriorly, the hippocampus was subpially disconnected from the amygdala, which lay anteromedial to the hippocampus. Posteriorly, the boundary of hippocampal resection was placed 2.5–3 cm from the anterior end of the hippocampus, where the hippocampus and parahippocampal gyrus were disconnected. The medial surface of the hippocampus was meticulously peeled away from the arachnoid covering the perimesencephalic cistern. This arachnoid layer was preserved in its entirety to prevent inadvertent injury to the midbrain and to keep blood from the resection site out of the cistern. In the same manner, the parahippocampal gyrus was dissected free from the leptomeninges covering the tentorial edge and the medial part of the temporal fossa.

- **Amygdalar resection**: Because there is no well-demarcated border between the amygdala and the basal ganglia, the upper limit of amygdalar resection must be carefully delineated to avoid iatrogenic injury to the globus pallidus. Different surgeons have used various landmarks to define the superior margin. We used an imaginary line connecting the inferior choroidal point and the limen insulae as this critical landmark. The amygdala was gently dissected from the surrounding structures using pressure-controlled suction and a bipolar coagulator. Dissection at the superior limit was performed cautiously to avoid penetrating injury to the basal ganglia. After circumferential dissection from the adjacent structures, the amygdala was gradually peeled away from the arachnoid covering the posterior cerebral artery and the oculomotor nerve.

As during hippocampectomy, this arachnoid plane should be preserved to prevent neurovascular injury and to keep blood from the surgical field out of the cistern. After complete amygdalohippocampectomy, the arachnoid membrane medial to both resected structures was clearly visualized.

- **Dynamic corridor and mesial temporal resection**: Entry into the temporal horn of the lateral ventricle and resection of the mesial temporal structures (amygdala, hippocampus, and parahippocampal gyrus) were achieved entirely through dynamic microsurgical maneuvers. The surgical corridor was maintained using gentle, transient micro-suction tips and bipolar forceps, completely avoiding static focal compression of adjacent eloquent parenchyma.
- **Hemostasis, dural closure, bone flap replacement, and scalp closure**: Hemostasis was meticulously achieved using bipolar electrocautery and absorbable oxidized cellulose. Any retained blood clots in the surgical bed or subarachnoid space were removed. The dura mater was closed in a watertight fashion to prevent cerebrospinal fluid leak; an artificial dural substitute was used when needed to cover any dural defect. The bone flap was repositioned and fixed with titanium plates and screws in good alignment for cosmetic outcome. The temporalis muscle was approximated, and a drainage catheter was placed beneath the scalp flap. The scalp was closed with non-absorbable sutures or staples.

### Statistical analysis

Statistical analysis was performed using Stata, release 13.0 (StataCorp LLC). Categorical variables are presented as number and percentage. Normally distributed continuous variables are reported as mean and standard deviation (SD), and nonnormally distributed variables as median and interquartile range (IQR). Between-group comparisons used chi-square or Fisher exact tests for categorical variables and independent t tests or Mann–Whitney *U* tests for continuous variables, as appropriate. The strength of association was expressed as the odds ratio (OR) with 95% confidence interval (CI). Statistical significance was defined as *P* < 0.05.

Variables with *P* < 0.20 in univariable analysis were entered into multivariable logistic regression models to identify independent predictors of postoperative seizure freedom (Engel Class Ia), with adjustment for clinically relevant confounders. Multivariable results are reported as ORs with 95% CIs. Discriminative performance and optimal cutoff values were assessed by receiver operating characteristic curve analysis. Seizure recurrence over time was analyzed using Kaplan–Meier curves with log-rank tests, and independent predictors were evaluated using Cox proportional hazards models, with results reported as hazard ratios with 95% CIs.

## Results

### Demographic characteristics (Table 1)

**Fig. 4:**
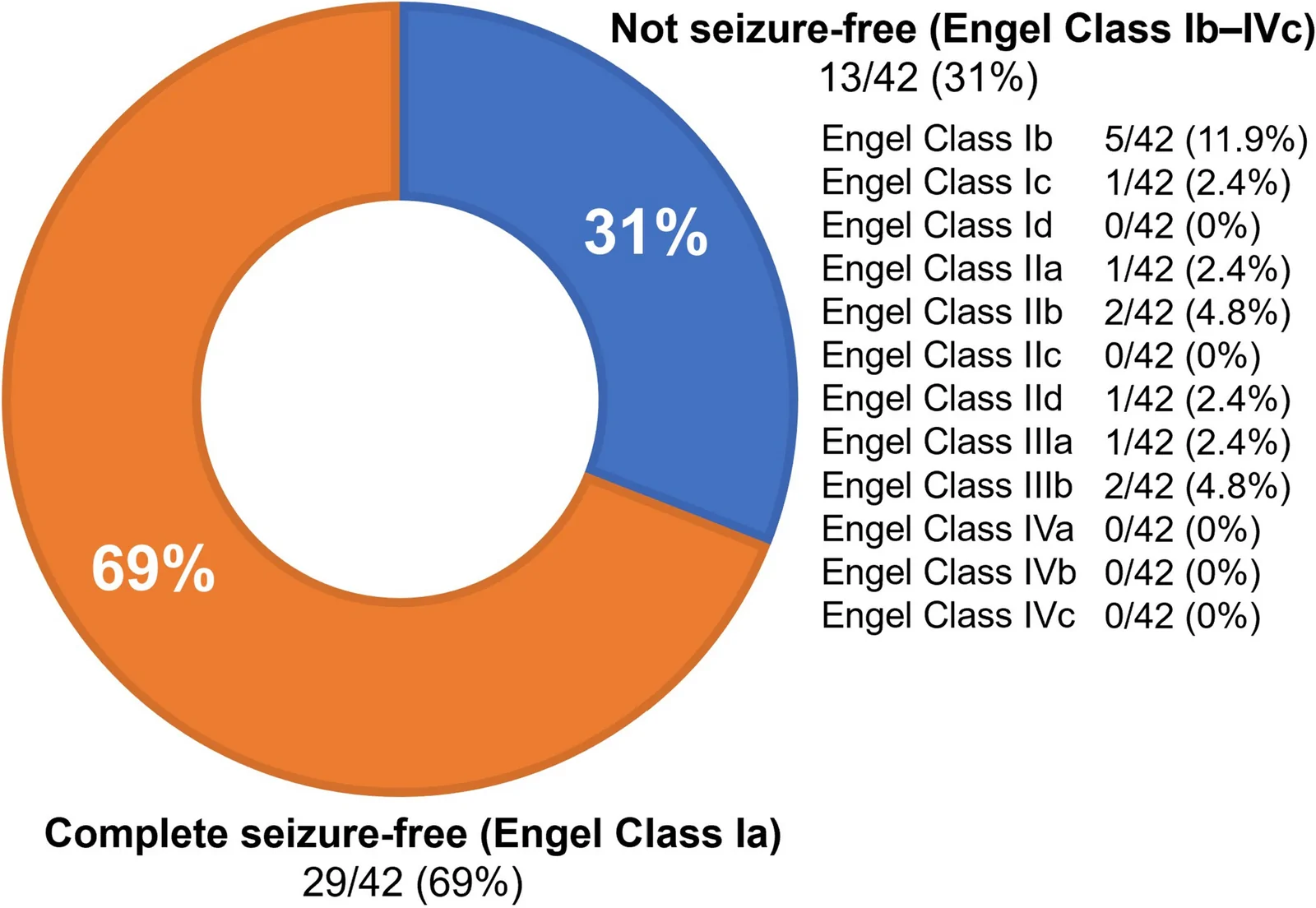
Postoperative seizure outcome assessed using the Engel classification. The pie chart shows the Engel-class distribution of seizure outcomes among the 42 patients. The orange segment represents 29 patients (69%) with complete seizure-free outcome (Engel Class Ia); the blue segment represents 13 patients (31%) without complete seizure-free outcome. The breakdown of Engel classes within the non–seizure-free group is shown to the right

During the eligible study period, 46 patients who underwent retractorless standard ATL were screened. Four cases were excluded because they had postoperative follow-up less than 24 months. Therefore, 42 patients were enrolled. They were categorized into 2 groups: 29 (69%) achieved complete postoperative seizure freedom (Engel Class Ia), and 13 (31%) did not (other Engel classes; Fig. 4). When seizure freedom from disabling seizures (Engel Class I) was considered, 35 of 42 patients (83.3%) achieved this outcome. The mean (SD) age at surgery was 30.8 (13.8) years. Twenty-three patients (54.8%) were female and 19 (45.2%) were male. The mean (SD) preoperative seizure duration was 14 (11.2) years. Mesial temporal sclerosis was the most common preoperative diagnosis (36 patients, 85.7%), followed by mesial temporal tumor (5, 11.9%) and non-lesional MTLE (1, 2.4%). Among epilepsy risk factors, a history of febrile convulsions was identified in 7 patients (16.7%) and central nervous system infection in 4 (9.5%). In comparisons between seizure outcome groups, female sex was the only demographic variable associated with seizure freedom (*P* = 0.049).

**Table 1:** Demographic characteristics of patients with and without postoperative seizure freedom

Variable		Overall (*n* = 42)	Comparison between groups with and without seizure freedom		
			Group with seizure freedom (*n* = 29)	Group without seizure freedom (*n* = 13)	*P* value
Age at surgery, y, mean (SD)		30.8 (13.8)	30.4 (13)	31.7 (15.8)	0.764
Sex, *n* (%)					
	Female	23 (54.8)	19 (65.5)	4 (30.8)	0.049^a^
	Male	19 (45.2)	10 (34.5)	9 (69.2)	
Preoperative duration of epilepsy, y, mean (SD)		14 (11.2)	13.7 (11.4)	14.8 (11.2)	0.682
Preoperative diagnosis, *n* (%)					
	Mesial temporal sclerosis	36 (85.7)	23 (79.3)	13 (100)	0.153
	Mesial temporal tumor	5 (11.9)	5 (17.2)	0 (0)	0.165
	Non-lesional MTLE	1 (2.4)	1 (3.5)	0 (0)	1.000
Location of lesion on MRI, *n* (%)					
	Hippocampus	36 (85.7)	23 (79.3)	13 (100)	0.153
	Amygdala	5 (11.9)	5 (17.2)	0 (0)	0.165
	No lesion	1 (2.4)	1 (3.5)	0 (0)	1.000
Risk factors for epilepsy, *n* (%)					
	CNS infection	4 (9.5)	2 (6.9)	2 (15.4)	0.576
	Febrile convulsion	7 (16.7)	6 (20.7)	1 (7.7)	0.405
	Asphyxia	1 (2.4)	1 (3.5)	0 (0)	1.000
	Traumatic brain injury	2 (4.8)	1 (3.5)	1 (7.7)	1.000
	Twins with drug-resistant epilepsy in the family	2 (4.8)	1 (3.5)	1 (7.7)	1.000

^a^ Indicates a statistically significant difference between the 2 groups

*CNS* central nervous system, *MRI* magnetic resonance imaging, *MTLE* mesial temporal lobe epilepsy, *n* number, *SD* standard deviation, *y* year(s)

### Presurgical evaluation (Table 2)

On seizure semiology, focal-onset seizures and seizures with impaired awareness were the most common types, occurring in 90.5% and 97.6% of patients, respectively. Approximately one-third of patients had secondary generalization after ictal onset. Thirty-seven patients (88.1%) had only 1 seizure type, whereas the remaining 5 (11.9%) had 2 types. The median seizure frequency was 1 per week (IQR, 1–2.8).

**Table 2:** Presurgical evaluation of patients with and without postoperative seizure freedom

Variable			Overall (*n* = 42)	Comparison between groups with and without seizure freedom		
				Group with seizure freedom (*n* = 29)	Group without seizure freedom (*n* = 13)	*P* value
Semiology, *n* (%)						
	Focal seizure		38 (90.5)	26 (89.7)	12 (92.3)	1.000
	Generalized seizure		4 (9.5)	3 (10.3)	1 (7.7)	
Ictal awareness, *n* (%)						
	Preserved awareness		1 (2.4)	0 (0)	1 (7.7)	0.310
	Impaired awareness		41 (97.6)	29 (100)	12 (92.3)	
Secondarily generalized seizure, *n* (%)						
	Present		14 (33.3)	8 (27.6)	6 (46.2)	0.298
	Absent		28 (66.7)	21 (72.4)	7 (53.8)	
Number of seizure types, *n* (%)						
	1 type		37 (88.1)	26 (89.7)	11 (84.6)	0.497
	2 types		5 (11.9)	3 (10.3)	2 (15.4)	
Seizure frequency, episodes/wk, median (IQR)			1 (1–2.8)	1 (1–2)	1 (0.9–5)	0.158
Location of interictal ED, *n* (%)						
	Ipsilateral temporal lobe		32 (76.2)	24 (82.8)	8 (61.5)	0.238
	Bilateral temporal lobes		10 (23.8)	5 (17.2)	5 (38.5)	
Location of onset of ictal ED, *n* (%)						
	Ipsilateral temporal lobe		27/37 (71.1)	19/24^b^ (79.2)	8/13 (61.5)	0.440
	Bilateral temporal lobes		10/37 (28.9)	5/24^b^ (20.8)	5/13 (38.5)	
Presence of TIRDA, *n* (%)			7/42 (16.7)	6/29 (20.7)	1/13 (7.7)	0.405
Concordance between interictal and ictal EEG findings, *n* (%)			24/37 (64.9)	17/24^b^ (63)	7/13 (53.8)	0.472
Lesion characteristic on MRI, *n* (%)						
	Hippocampal sclerosis		34 (81)	21 (72.4)	13 (100)	0.083
	Hippocampal atrophy		31 (73.8)	19 (65.5)	12 (92.3)	0.127
	Tumor		5 (11.9)	5 (17.2)	0 (0)	0.165
	Bilateral hippocampal sclerosis		3 (7.1)	1 (3.4)	2 (15.4)	0.222
Concordance between ictal EEG and side of lesion on MRI, *n* (%)			23/37 (62.2)	17/24^b^ (63)	6/13 (46.2)	0.171
Interictal SPECT showing hypoperfusion localized in the ipsilateral mesial temporal lobe, *n* (%)			28/37 (75.7)	21/25^c^ (84%)	7/12^d^ (58.3)	0.116
Wada test, *n* (%)						
		Nondominant language cerebral hemisphere ipsilateral to side of surgery or mesial temporal lesion	12/35 (34.3)	8/25^e^ (32)	4/10^f^ (40)	0.706
		Nondominant memory cerebral hemisphere ipsilateral to side of surgery or mesial temporal lesion	20/24 (83.3)	14/17^g^ (82.4)	6/7^h^ (85.7)	1.000
Number of preoperative ASDs, median (IQR)			3 (2.8–4)	3 (2–3.5)	4 (3–4)	0.044^a^

^a^ Indicates statistically significant difference between both groups

EEG studies:

^b^ Five of 29 patients in the seizure-free group were excluded from the ictal-ED analysis (*n* = 24). These 5 patients had no recorded seizures during multiple long-term video-EEG sessions, so their ictal EEG could not be obtained. All 5 underwent standard anterior temporal lobectomy based on concordant findings from semiology, interictal EEG, the MRI lesion, interictal SPECT, and the Wada test

Interictal SPECT:

^c^ Four of 29 patients in the seizure-free group did not undergo interictal SPECT (*n* = 25)

^d^ One of 13 patients in the non–seizure-free group did not undergo interictal SPECT (*n* = 12)

Wada test:

^e^ Four of 29 patients in the seizure-free group did not undergo the Wada test (*n* = 25)

^f^ Three of 13 patients in the non–seizure-free group did not undergo the Wada test (*n* = 10)

^g^ Among 25 in the group with seizure freedom who underwent the Wada test, the dominant side of memory function could not be lateralized in 7, and could not be evaluated in 1 due to serious confusion during the test (*n* = 17)

^h^ Among 10 in the group without seizure freedom who underwent the Wada test, the dominant side of memory function could not be lateralized in 3 (*n* = 7)

*ASD* antiseizure drug, *ED* epileptiform discharge, *EEG* electroencephalography, *IQR* interquartile range, *MRI* magnetic resonance imaging, *n* number, *SPECT* single-photon emission computed tomography, *TIRDA* temporal intermittent rhythmic delta activity, *wk* week

On EEG, most patients (76.2%) had interictal epileptiform discharges arising from the temporal lobe ipsilateral to the side of surgery. Ictal epileptiform discharges originated ipsilaterally in 71.1% of patients; the remaining 28.9% showed bilateral temporal onset. Temporal intermittent rhythmic delta activity was present in 16.7% of patients. No patient required intracranial EEG.

Cranial MRI of epileptogenic lesions showed that the most common abnormalities were hippocampal sclerosis (81%) and hippocampal atrophy (73.8%); bilateral hippocampal sclerosis was identified in 7.1% of patients. Neoplastic lesions were found in 11.9%. Concordance between the side of ictal epileptiform discharge and the side of the MRI lesion was present in 62.2% of analyzable cases.

Among patients who underwent interictal SPECT, 75.7% had hypoperfusion localized to the mesial temporal lobe ipsilateral to the operated side. Among the 35 patients who underwent the Wada test, 34.3% had a non-dominant language hemisphere on the side of surgery or mesial temporal lesion, and 83.3% had a non-dominant memory hemisphere on the same side. The median number of preoperative ASDs was 3 (IQR, 2.8–4).

Among presurgical evaluation variables, only the number of preoperative ASDs differed between seizure outcome groups. The seizure-free group received fewer preoperative ASDs than the non–seizure-free group (3 vs. 4; *P* = 0.044).

### Intraoperative and postoperative data and complications (Supplementary Table 1)

Of the 42 patients, 27 (64.3%) underwent left-sided surgery and 15 (35.7%) right-sided surgery. The mean (SD) operative time was 240.7 (30.2) min, and the mean (SD) operative blood loss was 253.6 (167.2) mL. The mean (SD) length of hospital stay was 8.8 (4.6) days. Seven postoperative complications occurred: bacterial meningitis (1 patient); skull osteomyelitis requiring surgical debridement and bone flap removal (1); hyponatremia from the syndrome of inappropriate antidiuretic hormone secretion (2); transient memory deficit (1); transient dysphasia (1); and subdural hygroma requiring a subduroperitoneal shunt (1). Early postoperative cranial CT showed no evidence of brain retraction injury, cerebral edema, or venous infarction. None of the patients developed an immediate post-operative seizure. No patient experienced permanent neurological disability or clinically significant visual field deficits as assessed by neurologists or ophthalmologists. No new seizure type or worsening of seizures was encountered during follow-up.

All patients underwent postoperative cranial MRI to assess the adequacy of temporal lobe resection. Adequate resection of the anterior temporal lobe and mesial temporal structures on MRI was achieved in 40 of 42 patients (95.2%). Of the 2 patients (4.8%) with inadequate resection, 1 had residual oligodendroglioma of the parahippocampal gyrus and underwent a second operation for tumor resection. This patient ultimately achieved long-term seizure freedom at the 80-month follow-up; the most recent MRI showed no residual or recurrent tumor. The other patient had residual amygdalar tissue on postoperative MRI but remained seizure-free from surgery through the most recent follow-up at 117 months and did not require reoperation.

Two patients required a second operation for seizure control. The first was the patient with residual parahippocampal oligodendroglioma described above. The second was a female patient with hippocampal sclerosis on the left side, which the Wada test had identified as her dominant language hemisphere. During the first few months after surgery her habitual seizures resolved completely, but they gradually returned to a disabling level. Re-evaluation of clinical, EEG, and imaging data showed that she was not a candidate for a second resection. She therefore underwent vagus nerve stimulation as the second procedure 26 months after retractorless standard ATL, with an Engel Class IIIb outcome at the 69-month follow-up.

For postoperative ASD adjustment, 13 of 29 seizure-free patients (44.8%) were able to discontinue ASD therapy long term. The mean (SD) time to ASD discontinuation in these 13 patients was 36.2 (16.3) months after surgery. Of the remaining seizure-free patients, 11 of 29 (37.9%) reduced their ASDs and 5 of 29 (17.3%) continued the same regimen as before surgery. Among the 29 seizure-free patients, 3 had their ASD regimens adjusted by neurologists in their hometowns (1 with reduction and 2 with no change). Among the 13 patients without seizure freedom, postoperative ASDs were increased in 5 (38.5%), unchanged in 3 (23%), and reduced in 5 (38.5%); none had ASD adjustment at outside hospitals. The median number of postoperative ASDs was 1.5 (IQR, 0–4). The mean (SD) postoperative follow-up was 66.3 (42) months.

Two postoperative variables were associated with seizure freedom. The seizure-free group had a shorter length of hospital stay than the non–seizure-free group (7.7 vs. 11.6 days; *P* = 0.028). Similarly, the seizure-free group required fewer postoperative ASDs than the non–seizure-free group (1 vs. 4; *P* = 0.001).

### Pathological characteristics (Supplementary Table 2)

The most common overall pathological diagnosis was hippocampal sclerosis (64.3% of cases), followed by focal cortical dysplasia/malformation of cortical development (38.1%). Hypoxic changes were identified in 16.7% of patients and neoplastic features in 9.5%. In the hippocampus, neuronal loss (64.3%) and reactive gliosis (45.2%) were the most common findings, whereas reactive gliosis (38.1%) and hypoxic changes (35.7%) predominated in the amygdala. The most common pathology of the anterior temporal cortex was focal cortical dysplasia/malformation of cortical development (40.5%). No association was identified between pathological features and seizure outcome.

### Univariable and multivariable analyses of factors associated with postoperative seizure freedom (Table 3)

In univariable analysis, 5 variables were associated with postoperative seizure freedom:

**Table 3:** Univariable and multivariable analyses of factors associated with postoperative seizure freedom

Variable		Unadjusted OR (95% CI)	*P* value	Adjusted OR (95% CI)	*P* value
Sex					
	Male (reference)	Reference		Reference	
	Female	4.28 (1.05–17.42)	0.043^a^	1.54 (0.13–18.16)	0.732
Preoperative seizure frequency					
	≥ 3 times/wk	Reference		Reference	
	< 3 times/wk	5.36 (1.17–24.43)	0.030^a^	24.21 (1.46–402.13)	0.026^a^
Concordance between ictal EEG and side of lesion on MRI					
	Discordant	Reference		Reference	
	Concordant	2.83 (0.51–8.45)	0.022^a^	6.93 (0.52–92.30)	0.143
Interictal SPECT showing hypoperfusion localized in the ipsilateral mesial temporal lobe					
	Not localized hypoperfusion	Reference		Reference	
	Localized hypoperfusion	1.63 (0.23–11.45)	0.305	12.10 (0.30–489.41)	0.187
Number of preoperative ASD					
	≥ 4	Reference		Reference	
	< 4	7.07 (1.65–30.23)	0.008^a^	3.52 (0.36–34.05)	0.278
Side of surgery					
	Right	Reference		Reference	
	Left	3.06 (0.79–11.94)	0.107	1.42 (0.12–17.48)	0.783
Length of hospital stay					
	≥ 11 d	Reference		Reference	
	< 11 d	7.29 (1.60–33.26)	0.010^a^	1.56 (0.17–13.92)	0.692
Pathological finding in anterior temporal cortex					
	Unremarkable finding	Reference		Reference	
	Apparent pathological finding	3.85 (0.72–20.62)	0.115	3.66 (0.21–62.47)	0.370

^a^ Indicates a statistically significant association with postoperative seizure freedom

Variables with *P* < 0.2 in the univariable analysis were included in the multivariable model: sex; preoperative diagnosis (mesial temporal sclerosis, mesial temporal tumor); location of the lesion (hippocampus, amygdala); preoperative seizure frequency; lesion characteristics on MRI (hippocampal sclerosis, hippocampal atrophy, tumor); concordance between ictal EEG and side of the MRI lesion; interictal SPECT showing hypoperfusion localized to the mesial temporal lobe ipsilateral to the surgical side; number of preoperative ASDs; side of surgery; length of hospital stay; amygdalar pathology showing tumor; and anterior temporal cortex pathology showing reactive gliosis or unremarkable findings

Odds ratios could not be calculated for the following variables: preoperative diagnosis (mesial temporal sclerosis, mesial temporal tumor); location of the lesion (hippocampus, amygdala); lesion characteristics on MRI (hippocampal sclerosis, hippocampal atrophy, tumor); amygdalar pathology showing tumor; and anterior temporal cortex pathology showing reactive gliosis

*ASD* antiseizure drug, *CI* confidence interval, *d* day(s), *EEG* electroencephalography, *MRI* magnetic resonance imaging, *OR* odds ratio, *SPECT* single-photon emission computed tomography, *wk* week

- female sex (OR, 4.28; 95% CI, 1.05–17.42; *P* = 0.043);
- preoperative seizure frequency < 3 times per week (OR, 5.36; 95% CI, 1.17–24.43; *P* = 0.030);
- concordance between the side of ictal epileptiform discharge and the MRI lesion (OR, 2.83; 95% CI, 0.51–8.45; *P* = 0.022);
- fewer than 4 preoperative ASDs (OR, 7.07; 95% CI, 1.65–30.23; *P* = 0.008); and
- length of hospital stay < 11 days (OR, 7.29; 95% CI, 1.60–33.26; *P* = 0.010).

In multivariable logistic regression, preoperative seizure frequency < 3 times per week was the only independent predictor of long-term postoperative seizure freedom (OR, 24.21; 95% CI, 1.46–402.13; *P* = 0.026).

Receiver operating characteristic analysis showed that preoperative seizure frequency had modest discriminative ability for predicting postoperative seizure outcome, with an area under the curve of 0.65. A cutoff of ≥ 3 seizures per week was associated with failure to achieve seizure freedom, with a sensitivity of 46.2% and a specificity of 82.8% (Supplementary Fig. 2).

**Fig. 5:**
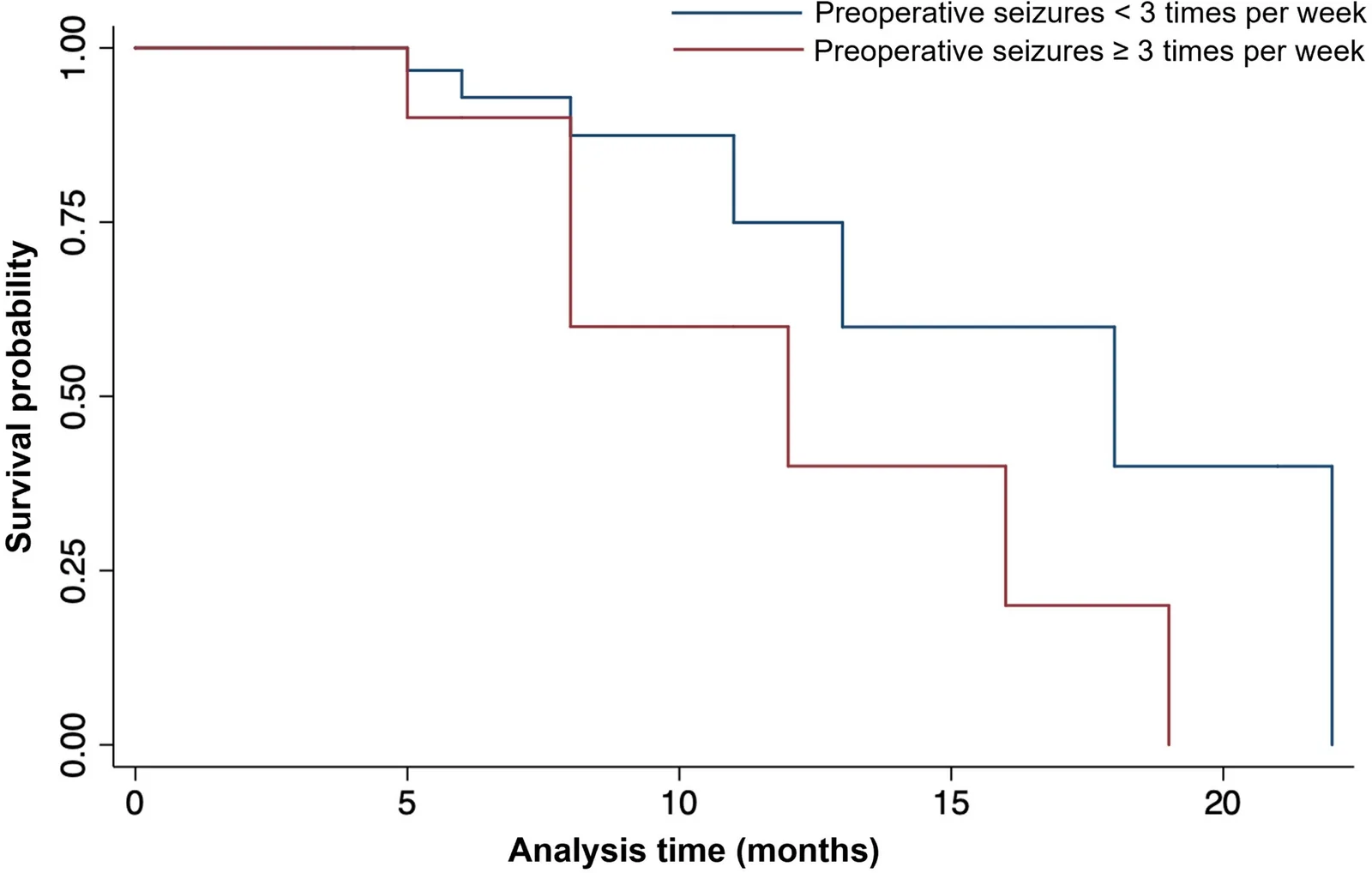
Kaplan–Meier analysis of seizure freedom stratified by preoperative seizure frequency. The adjusted Cox proportional hazards model showed that a preoperative seizure frequency ≥ 3 seizures per week was associated with an increased risk of failure to achieve seizure freedom (adjusted hazard ratio, 7.34; 95% CI, 1.08–50.04; *P* = 0.042). The adjusted variables in the adjusted Cox proportional hazards model included gender, concordance between ictal EEG and MRI lesion side, interictal SPECT showing hypoperfusion localized to the ipsilateral mesial temporal lobe, number of preoperative ASDs, surgical side, and length of hospital stay. Abbreviation: ASD, antiseizure drug; CI, confidence interval; EEG, electroencephalography; MRI, magnetic resonance imaging; SPECT, single-photon emission computed tomography

Kaplan–Meier survival analysis showed a markedly lower probability of postoperative seizure freedom in patients with a higher preoperative seizure frequency (≥ 3 times per week) than in those with < 3 seizures per week. Patients with ≥ 3 seizures per week showed an earlier and more rapid decline in seizure-free survival during follow-up. In the unadjusted Cox proportional hazards model, ≥ 3 seizures per week was associated with an increased risk of failure to achieve seizure freedom. However, this association did not reach statistical significance (unadjusted hazard ratio, 2.47; 95% CI, 0.79–7.74; *P* = 0.121). After adjustment for relevant confounders, a higher preoperative seizure frequency (≥ 3 times per week) emerged as an independent predictor of failure to achieve seizure freedom. These patients had a substantially increased hazard relative to those with < 3 seizures per week (adjusted hazard ratio, 7.34; 95% CI, 1.08–50.04; *P* = 0.042; Fig. 5).

## Discussion

Brain retractors are common instruments used in intracranial procedures to facilitate operative access by applying focal pressure to the brain surface. Inadvertently excessive retraction can produce focal cerebral ischemia, cerebral edema, and parenchymal injury, leading to postoperative complications [16, 33–37]. Postsurgical brain edema, in particular, contributes to early postoperative seizures by raising intracranial pressure and disrupting neuronal stability [38]. Various strategies have been used to reduce retraction, preserve cortical perfusion, and limit the risk of venous congestion, including gravity-assisted positioning, handheld dynamic retractors, and precise arachnoid dissection that maintains operative exposure without fixed-blade retractors [39].

The retractorless technique has been increasingly proposed for neurosurgical operations in recent years [35, 36]. The principal rationale is the minimization or avoidance of retraction injury [40]. For example, the retractorless technique has been used in the transcallosal–interforniceal approach for resection of third ventricular tumors. Gross total resection was achieved in 80.6% of patients, with no evidence of brain retraction injury or edema [27]. These findings align with evidence from skull base procedures showing low rates of temporal lobe injury (approximately 3%–5%), even in complex meningioma resections [40].

Clinical data show that retractorless techniques across a range of complex neurosurgical conditions achieve successful treatment in approximately 94.25% of cases, with fewer complications [39]. Broader cranial surgery data support reduced morbidity, positioning retractorless methods as a viable option for selected epilepsy cases with favorable anatomy [40]. Challenges include a demanding learning curve for less experienced surgeons and limitations when targeting deep lesions, where microsurgical expertise and neuromonitoring are required [39].

Because the retractorless technique reduces retraction-related brain injury and subsequent edema and may also limit the risk of new epileptogenic foci induced by cortical injury, we have adopted it for various surgical procedures for intractable epilepsy. The most frequent application has been standard ATL for the treatment of drug-resistant MTLE.

### Retractorless standard ATL and seizure outcomes

In our study, retractorless standard ATL produced favorable seizure outcomes in the majority of patients. Approximately 70% achieved long-term complete seizure freedom (Engel Class Ia). Most patients who did not achieve seizure freedom fell into low-severity Engel categories such as Class Ib. When the broader Engel Class I outcome was considered, 35 of 42 patients (83.3%) reached this level. These results indicate that the retractorless procedure achieved seizure freedom in a large proportion of patients and substantially reduced seizure severity in the remainder.

The seizure freedom rate in our study was consistent with previously reported outcomes after MTLE surgery. Prior studies have shown Engel Class I outcomes in approximately 60%–80% of patients after ATL or selective amygdalohippocampectomy [41–43]. In a large series of patients with MTLE treated by ATL, Engel Class I outcomes were achieved in 78.6% of cases, with durable long-term seizure control [44]. Systematic reviews and meta-analyses report pooled Engel Class I rates after ATL of approximately 64%–73% [45–48], supporting the validity of our findings. The landmark randomized controlled trial of standard ATL for temporal lobe epilepsy reported a 1-year cumulative proportion of patients free from seizures impairing awareness of 58% [49]. Large retrospective analyses of conventional resective surgery for MTLE typically report Engel Class I rates of 58%–73% [50]. In cohorts limited to hippocampal sclerosis, standard ATL has achieved Engel Class I rates of approximately 73.6%–78.6% [50, 51]. Longitudinal studies have shown that short-term seizure freedom after standard ATL can reach 82%–86%, with modest decline over 5–10 years of follow-up [51, 52]. The 83.3% Engel Class I rate in our series therefore lies in the upper tier of results reported for conventional surgical techniques.

The predominance of Engel Class Ib among our non–seizure-free patients aligns with previous reports indicating that postoperative seizures tend to be less frequent and less disabling than preoperative seizures [42, 53]. Residual seizure activity has been attributed to incomplete resection of the epileptogenic zone, dual pathology, or widespread epileptogenic networks beyond the mesial temporal structures. These factors have been consistently associated with less favorable outcomes after temporal lobe surgery [54–56].

Overall, our findings indicate that retractorless standard ATL provides a high rate of seizure control. The high proportion of favorable outcomes supports its role as an effective surgical option for drug-resistant MTLE, and the distribution of non–seizure-free outcomes indicates meaningful seizure reduction even in patients who do not achieve complete seizure freedom.

### Safety, complications, adequate resection, and reoperation

Our study showed that retractorless surgery for MTLE has a favorable safety profile. In conventional MTLE surgery, fixed spatulas are typically introduced during initial elevation of the temporal lobe to visualize the skull base, and again during deep dissection of the mesial temporal structures. Although these instruments provide a stable view, they exert continuous pressure on the brain parenchyma, which can produce localized ischemia or postoperative edema. By contrast, the retractorless approach replaces this static tension with a dynamic strategy centered on brain relaxation and gravity.

The retractorless technique begins with strategic head positioning that allows the temporal lobe to fall naturally away from the surgical corridor, producing a relaxed brain with sufficient working space for visualization. Throughout the procedure we use dynamic retraction, in which the surgeon’s suction tip provides only transient, gentle support to tissue at the point of dissection rather than continuous pressure on a single area. This approach keeps the surgical field unobstructed and preserves cortical integrity, as evidenced by the absence of retraction-related complications in our series.

These observations are consistent with previous reports showing that complex vascular and skull base surgery can be performed safely with minimal fixed retraction or with a retractorless technique. This approach reduces the risk of retraction-related cortical injury, venous infarction, and postoperative edema [26, 37, 39, 40]. Techniques emphasizing gravity-assisted exposure and dynamic retraction have been associated with lower rates of approach-related morbidity while preserving adequate visualization of the mesial temporal structures [26]. These studies indicate that meticulous microsurgical technique, rather than the use of fixed retractors, is the primary determinant of surgical safety. Our findings extend this concept, suggesting that retractorless approaches can maintain equivalent surgical completeness without increasing perioperative risk in anteromesial temporal resection for epilepsy.

The complications observed in our series included those typically expected after ATL, such as transient dysphasia and transient memory deficit. Other complications were those that may occur in any intracranial surgery, such as hyponatremia, bacterial meningitis, skull osteomyelitis, and subdural hygroma. None were related to intraoperative brain retraction injury. Our complication profile largely mirrors published benchmarks, reinforcing that ATL has a manageable safety margin. Although the literature reports low risks of meningitis (0.5%–2%) and osteomyelitis (< 0.5%), we encountered 1 case of each, equating to approximately 2.4% in our 42-patient cohort. This modest elevation is more likely a reflection of the small sample size than a departure from surgical standards [57, 58]. Transient memory deficit and transient language deficit each occurred in 2.4% of our patients, well below the 20%–40% risk of verbal memory decline reported in the literature, particularly for dominant-hemisphere surgery [57]. We routinely used the Wada test to determine language and memory lateralization, addressing the rigorous preoperative testing recommended in the literature to mitigate such cognitive risks [59].

All patients underwent postoperative cranial MRI to evaluate the resected area. Imaging confirmed adequate resection of the anterior temporal lobe and mesial temporal structures in 95.2% of patients, with no statistically significant difference in resection adequacy between the seizure-free and non–seizure-free groups. The need for reoperation was uncommon and comparable between groups. These findings suggest that differences in seizure outcome were unlikely to reflect technical inadequacy or insufficient surgical exposure, supporting the procedural reliability of the retractorless approach. The absence of increased reoperation rates, together with adequate resection on postoperative imaging, is consistent with large surgical series of conventional ATL and selective amygdalohippocampectomy. These series have reported low rates of major complications and revision surgery [49, 60].

The high technical success rate in our study—adequate resection in 95.2% of patients—is consistent with reports that surgical management yields superior outcomes to medical therapy alone. Even in the 2 patients with inadequate initial resection, long-term seizure freedom was still achievable, with follow-up extending to 80–117 months, through either reoperation or continued monitoring. These findings support the literature reporting that 84%–97% of patients endorse the procedure long term because of substantial quality-of-life gains [61]. Although complications such as the syndrome of inappropriate antidiuretic hormone secretion or infection can occur, they are typically resolvable with medical or surgical management.

Patients who did not achieve seizure freedom required substantially more postoperative antiseizure medications in the long term. This likely reflects greater underlying epileptogenic complexity rather than surgical factors, because imaging-confirmed resection adequacy was similar between groups. A previous study has similarly reported that persistent seizures after temporal lobe surgery are associated with a higher antiseizure medication burden despite technically successful resections [59].

Taken together, these findings support the safety of retractorless standard ATL, demonstrating adequate resection and low reoperation rates. Combined with favorable seizure outcomes, this approach represents a safe and effective surgical option for patients with drug-resistant MTLE.

### Factors associated with postoperative seizure outcomes

In our study, multivariable logistic regression identified a lower preoperative seizure frequency (< 3 seizures per week) as the only independent predictor of postoperative seizure freedom after retractorless standard ATL. Although several variables—including sex, EEG–MRI concordance, number of preoperative ASDs, and length of hospital stay—were associated with seizure freedom in univariable analyses, these associations did not persist after adjustment. This finding highlights preoperative seizure burden as the dominant determinant of postoperative seizure outcome in this cohort.

### Lower seizure frequency and favorable surgical outcome

Previous studies have shown that a higher preoperative seizure frequency or longer duration of epilepsy reflects a more extensive or aggressive epileptogenic network that is less amenable to focal resective surgery [59, 62–64]. In large multicenter cohorts, patients with fewer preoperative seizures have consistently shown higher rates of seizure freedom, supporting seizure burden as a surrogate marker of disease severity and network complexity [59, 64]. Our findings extend these observations to patients undergoing retractorless standard ATL, reinforcing preoperative seizure frequency as a robust prognostic indicator across surgical techniques.

Although concordance between ictal EEG and MRI lesion laterality was a notable factor associated with seizure freedom in unadjusted analysis, it did not retain independent significance in the multivariable model. This finding contrasts with earlier studies reporting improved outcomes in patients with well-localized, concordant presurgical data [57, 63]. The loss of significance after adjustment in our study suggests that, in the presence of clearly defined mesial temporal pathology and adequate resection, seizure burden may outweigh other localization-related factors in determining postoperative seizure control. Similarly, the number of preoperative antiseizure medications was associated with seizure freedom in univariable analysis but was not an independent predictor after adjustment. Prior studies have reported that polytherapy often reflects pharmacoresistance and longer disease duration, both of which are linked to poorer outcomes [64, 65]. However, our results suggest that the functional expression of disease severity—manifested as seizure frequency—may be a more direct and clinically relevant predictor than medication burden alone.

### Strengths

A key strength of this study is that all surgical candidates were carefully evaluated and selected by a multidisciplinary team of epilepsy specialists at our institution. All procedures were performed by a single neurosurgeon (B.S.) using an identical and consistent surgical technique, which avoided inter-operator variability and likely reduced intra-operative variability. To our knowledge, retractorless standard ATL has not previously been described in the literature, although some surgeons may use this strategy without reporting it.

### Limitations

Several limitations should be acknowledged. First, the retrospective design may have introduced confounding. However, a complete postoperative follow-up rate and comprehensive preoperative and postoperative data collection should help mitigate this risk. Second, the overall postoperative seizure-free rate of 69% was relatively lower than that reported in some prior studies. This may be explained, in part, by the inclusion of 3 patients with bilateral hippocampal sclerosis who deliberately underwent surgery as a palliative procedure aimed at reducing seizure burden rather than achieving seizure freedom. Their inclusion may have led to an underestimation of the true seizure-free rate in patients undergoing surgery with curative intent. Moreover, our seizure-free analysis focused on patients achieving Engel Class Ia (completely free of disabling seizures), accounting for the 69% rate. When the broader Engel Class I outcome is considered, the rate rises to 83.3%, which is comparable to the high success rates reported in most studies.

Third, no direct comparison between the retractorless and conventional techniques was performed, because the vast majority of standard ATLs at our center are now performed using the retractorless approach. The number of patients undergoing each technique is therefore highly imbalanced, making direct comparison methodologically inappropriate. Consequently, our findings cannot definitively confirm the absolute superiority or non-inferiority of the retractorless technique regarding operative time, localized tissue contusion, or seizure freedom compared to conventional retractors. The future study should compare surgical outcomes and complications of ATL between the well-matched groups with and without the use of a brain retractor. Multi-institutional studies with well-matched comparative arms are necessary to validate these comparative endpoints.

Fourth, compared to other surgical strategies for MTLE, such as keyhole temporal lobectomy or selective amygdalohippocampectomy, ATL employing the retractorless technique required a comparatively greater craniotomy size and more extensive lateral temporal cortex resection. With a larger craniotomy size and cortical resection, bleeding should be cautious, particularly in patients taking sodium valproate. Nevertheless, as stated in the section on surgical procedures, we carefully restricted the posterior boundary of neocortical temporal resection. The mesial temporal structures were clearly visible when the temporal neocortex was sufficiently removed, which may enhance the suitability of mesial temporal resection.

Fifth, owing to the distance between some patients’ hometowns and our hospital, 3 patients with long-term seizure-free outcomes were transferred to neurologists closer to home for postoperative ASD management. None of the patients was able to discontinue medication. A major reason was that these neurologists were reluctant to reduce or discontinue ASDs despite many years of seizure freedom after surgery. This may have led to an underestimation of the rate of postoperative ASD cessation in our seizure-free patients.

Finally, this study was conducted at a tertiary referral hospital, so generalizability to other populations may be limited. Retractorless neurosurgical techniques also require dedicated practice and a learning curve, and some surgeons may not be familiar with this strategy. Reproducibility of the surgical outcome may therefore be restricted.

## Conclusions

In this descriptive study of a surgical approach, retractorless standard ATL provides effective long-term seizure control in patients with drug-resistant MTLE. In our cohort, 69% of patients achieved complete seizure freedom (Engel Class Ia), 83.3% achieved freedom from disabling seizures (Engel Class I), and most non–seizure-free patients experienced substantial seizure reduction. Surgical safety was favorable, with a high rate of adequate resection, no complications related to brain retraction injury, and a low reoperation rate. Of all variables evaluated, a lower preoperative seizure frequency (< 3 seizures per week) was the only independent predictor of postoperative seizure freedom, highlighting seizure burden as the dominant determinant of postoperative seizure outcome.

## Supplementary Information

Below is the link to the electronic supplementary material.

Supplementary Fig. 6 Plan of the surgical incision and temporal craniotomy for retractorless standard anterior temporal lobectomy, left side. The patient’s head is fixed in a 3-point Mayfield head holder. The question-mark incision is marked by the blue line. Three burr holes are placed at the frontal keyhole (circle 1), the inferior temporal region (circle 2), and the superior temporal line (circle 3). The outline of the temporal craniotomy is shown by the red circle (JPEG 2.34 MB)

Supplementary Fig. 7 Receiver operating characteristic (ROC) analysis of the discriminative value of preoperative seizure frequency for predicting postoperative seizure outcome. The area under the ROC curve (AuROC) is 0.65 (95% CI, 0.46–0.85). A cutoff of ≥ 3 seizures per week was associated with failure to achieve postoperative seizure freedom (sensitivity, 46.2%; specificity, 82.8%). Abbreviations: AuROC, area under the receiver operating characteristic curve; CI, confidence interval; ROC, receiver operating characteristic (JPEG 343 KB)

Supplementary Material 3 (DOCX 17.8 KB)

Supplementary Material 4 (DOCX 17.9 KB)

## Data Availability

No datasets were generated or analysed during the current study.

## Abbreviations

ASD: Antiseizure Drug
ATL: Anterior Temporal Lobectomy
CI: Confidence Interval
CT: Computed Tomography
EEG: Electroencephalography
IQR: Interquartile Range
MRI: Magnetic Resonance Imaging
MTLE: Mesial Temporal Lobe Epilepsy
OR: Odds Ratio
SD: Standard Deviation
