# Supplementary material for: Standard anterior temporal lobectomy using a retractorless technique in patients with drug-resistant mesial temporal lobe epilepsy: long-term seizure outcomes and predictors of postoperative seizure freedom
Source: Neurosurg Rev. 2026 Nov 3;49(1):630. doi: 10.1007/s10143-026-04555-9 (PMC13731526; doi:10.1007/s10143-026-04555-9)
Supplement: Supplementary file 1 [file 10143_2026_4555_MOESM3_ESM.docx]

**Supplementary Table 1** Intraoperative and postoperative variables in patients with and without postoperative seizure freedom

| **Variable** | | **Overall (*n* = 42)** | **Comparison between groups with and without seizure freedom** | | |
| --- | --- | --- | --- | --- | --- |
|  |  |  | **Group with seizure freedom (*n* = 29)** | **Group without seizure freedom (*n* = 13)** | ***P* value** |
| Surgical side, *n* (%) | |  |  |  |  |
|  | Left side | 27 (64.3) | 21 (72.4) | 6 (46.2) | 0.163 |
|  | Right side | 15 (35.7) | 8 (27.6) | 7 (53.8) |  |
| Operative time, min, mean (SD) | | 240.7 (30.2) | 240.9 (30.9) | 240.4 (29.8) | 0.960 |
| Blood loss, mL, mean (SD) | | 253.6 (167.2) | 236.9 (161.3) | 290.8 (180.6) | 0.347 |
| Length of hospital stay, d, mean (SD) | | 8.8 (4.6) | 7.7 (3.4) | 11.6 (5.7) | 0.028^a^ |
| Presence of complications, *n* (%) | | 7 (16.7) | 5 (17.2) | 2 (15.4) | 1.000 |
| Complication, *n* (%) | |  |  |  |  |
|  | Infection | 2 (4.8) | 1^b^ (3.4) | 1^c^ (7.7) | 1.000 |
|  | SIADH | 2 (4.8) | 2 (6.9) | 0 (0) | 0.562 |
|  | Transient memory loss | 1 (2.4) | 1 (3.4) | 0 (0) | 1.000 |
|  | Transient dysphasia | 1 (2.4) | 1 (3.4) | 0 (0) | 1.000 |
|  | Subdural hygroma | 1 (2.4) | 0 (0) | 1^d^ (7.7) | 0.310 |
| Adequate resection of anterior temporal lobe and mesial temporal structures on postoperative MRI, *n* (%) | | 40 (95.2) | 27^e,f^ (93.1) | 13 (100) | 0.562 |
| Requirement of second operation, *n* (%) | | 2 (4.8) | 1^f^ (3.4) | 1^g^ (7.7) | 1.000 |
| Number of long-term postoperative AED, median (IQR) | | 1.5 (0–4) | 1 (0–3) | 4 (2.5–4) | 0.001^a^ |
| Postoperative follow-up, mo, mean (SD) | | 66.3 (42) | 69.1 (44) | 60.1 (38.8) | 0.562 |

^a^ Indicates a statistically significant difference between the 2 groups.
^b^ The patient developed postoperative bacterial meningitis.
^c^ The patient developed postoperative skull osteomyelitis, requiring removal of the bone flap.
^d^ An older patient had persistent postoperative subdural hygroma, requiring a subduroperitoneal shunting procedure.
^e^ The patient had residual amygdalar tissue on postoperative MRI but achieved a long-term seizure-free outcome (Engel Class Ia).
^f^ The patient had a residual tumor in the parahippocampal gyrus, requiring a second operation for tumor resection.
^g^ The patient underwent vagus nerve stimulation after retractorless standard anterior temporal lobectomy to control residual seizures.
**Abbreviations:** AED, antiepileptic drug; d, day(s); IQR, interquartile range; min, minute(s); mL, milliliter(s); mo, month(s); MRI, magnetic resonance imaging; *n*, number; SD, standard deviation; SIADH, syndrome of inappropriate antidiuretic hormone secretion.
