# Supplementary material for: Standard anterior temporal lobectomy using a retractorless technique in patients with drug-resistant mesial temporal lobe epilepsy: long-term seizure outcomes and predictors of postoperative seizure freedom
Source: Neurosurg Rev. 2026 Nov 3;49(1):630. doi: 10.1007/s10143-026-04555-9 (PMC13731526; doi:10.1007/s10143-026-04555-9)
Supplement: Supplementary file 2 [file 10143_2026_4555_MOESM4_ESM.docx]

**Supplementary Table 2** Pathological features of patients with and without postoperative seizure freedom

| **Variable** | | **Overall (*n* = 42)** | **Comparison between groups with and without seizure freedom** | | |
| --- | --- | --- | --- | --- | --- |
|  |  |  | **Group with seizure freedom (*n* = 29)** | **Group without seizure freedom (*n* = 13)** | ***P* value** |
| Pathological diagnosis^a^, *n* (%) | |  |  |  |  |
|  | Hippocampal sclerosis | 27 (64.3) | 18 (62.1) | 9 (69.2) | 0.739 |
|  | FCD/MCD | 16 (38.1) | 12 (41.4) | 4 (30.8) | 0.733 |
|  | Hypoxic change | 7 (16.7) | 5 (17.2) | 2 (15.4) | 1.000 |
|  | Tumor (DNET, oligoastrocytoma) | 4 (9.5) | 4 (13.8) | 0 (0) | 0.293 |
| Hippocampal pathology^b^, *n* (%) | |  |  |  |  |
|  | Neuronal loss | 27 (64.3) | 18 (62.1) | 9 (69.2) | 0.739 |
|  | FCD/MCD | 0 (0) | 0 (0) | 0 (0) | 1.000 |
|  | Reactive gliosis | 19 (45.2) | 13 (44.8) | 6 (46.2) | 1.000 |
|  | Hypoxic change | 11 (26.2) | 7 (24.1) | 4 (30.8) | 0.713 |
|  | Tumor (DNET) | 3 (7.1) | 3 (10.3) | 0 (0) | 0.540 |
|  | Unremarkable | 1 (2.4) | 1 (3.4) | 0 (0) | 1.000 |
| Amygdalar pathology^b^, *n* (%) | |  |  |  |  |
|  | Neuronal loss | 12 (28.6) | 8 (27.6) | 4 (30.8) | 1.000 |
|  | FCD/MCD | 0 (0) | 0 (0) | 0 (0) | 1.000 |
|  | Reactive gliosis | 16 (38.1) | 11 (37.9) | 5 (38.5) | 1.000 |
|  | Hypoxic change | 15 (35.7) | 9 (31) | 6 (46.2) | 0.488 |
|  | Tumor (DNET, oligoastrocytoma) | 5 (11.9) | 5 (17.2) | 0 (0) | 0.165 |
|  | Unremarkable | 4 (9.5) | 3 (10.3) | 1 (7.7) | 1.000 |
| Anterior temporal cortex pathology^b^, *n* (%) | |  |  |  |  |
|  | Neuronal loss | 1 (2.4) | 1 (3.4) | 0 (0) | 1.000 |
|  | FCD/MCD | 17 (40.5) | 12 (41.4) | 5 (38.5) | 1.000 |
|  | Reactive gliosis | 5 (11.9) | 5 (17.2) | 0 (0) | 0.165 |
|  | Hypoxic change | 14 (33.3) | 10 (34.5) | 4 (30.8) | 1.000 |
|  | Tumor (DNET) | 1 (2.4) | 1 (3.4) | 0 (0) | 1.000 |
|  | Unremarkable | 7 (16.7) | 3 (10.3) | 4 (30.8) | 0.176 |

No variable showed a statistically significant difference between the two groups in **Supplementary Table 2.**
^a^ A patient could have more than one pathological diagnosis.
^b^ A specific structure could show more than one pathological finding.
**Abbreviations:** DNET, dysembryoplastic neuroepithelial tumor; FCD, focal cortical dysplasia; MCD, malformation of cortical development; *n*, number.
